# Motion-Induced Blindness and Troxler Fading: Common and Different Mechanisms

**DOI:** 10.1371/journal.pone.0092894

**Published:** 2014-03-21

**Authors:** Yoram S. Bonneh, Tobias H. Donner, Alexander Cooperman, David J. Heeger, Dov Sagi

**Affiliations:** 1 Department of Human Biology, University of Haifa, Haifa, Israel; 2 Department of Neurobiology, The Weizmann Institute of Science, Rehovot, Israel; 3 Department of Psychology, University of Amsterdam, Amsterdam, The Netherlands; 4 Cognitive Science Center, University of Amsterdam, Amsterdam, The Netherlands; 5 Bernstein Center for Computational Neuroscience, Charité-Universitätsmedizin, Berlin, Germany; 6 Department of Psychology and Center for Neural Science, New York University, New York, New York, United States of America; The Ohio State University, Center for Cognitive and Brain Sciences, Center for Cognitive and Behavioral Brain Imaging, United States of America

## Abstract

Extended stabilization of gaze leads to disappearance of dim visual targets presented peripherally. This phenomenon, known as Troxler fading, is thought to result from neuronal adaptation. Intense targets also disappear intermittently when surrounded by a moving pattern (the “mask”), a phenomenon known as motion-induced blindness (MIB). The similar phenomenology and dynamics of these disappearances may suggest that also MIB is, likewise, solely due to adaptation, which may be amplified by the presence of the mask. Here we directly compared the dependence of both phenomena on target contrast. Observers reported the disappearance and reappearance of a target of varying intensity (contrast levels: 8%–80%). MIB was induced by adding a mask that moved at one of various different speeds. The results revealed a lawful effect of contrast in both MIB and Troxler fading, but with opposite trends. Increasing target contrast increased (doubled) the rate of disappearance events for MIB, but decreased the disappearance rate to half in Troxler fading. The target mean invisible period decreased equally strongly with target contrast in MIB and in Troxler fading. The results suggest that both MIB and Troxler are equally affected by contrast adaptation, but that the rate of MIB is governed by an additional mechanism, possibly involving antagonistic processes between neuronal populations processing target and mask. Our results link MIB to other bi-stable visual phenomena that involve neuronal competition (such as binocular rivalry), which exhibit an analogous dependency on the strength of the competing stimulus components.

## Introduction

When a global moving pattern surrounds a high-contrast stationary or slowly moving pattern, this pattern disappears and reappears periodically, a phenomenon called ‘motion-induced blindness’ (MIB) [1]. MIB thus belongs to a class of multistable phenomena in which an unchanging stimulus generates alternating perceptual states, such as the Necker cube, ambiguous structure from motion and binocular-rivalry [2]. However, MIB is also a “visual disappearance” or fading phenomena, in which otherwise salient visible stimuli disappear from awareness, as if erased in front of the observer's eyes. These include binocular rivalry [2], “generalized flash suppression” [3], artificial scotoma [4], Troxler fading [5] and the related “scene fading” effect [6]. The interest in MIB and the other disappearance effects stems from the all-or-none nature of the illusory disappearance, which could be useful for identifying the neural correlates of consciousness [7] and the intrinsic mechanisms underlying perceptual organization [2], [8].

Current evidence shows that MIB is not solely determined by low-level sensory suppression or adaptation and is not caused by a shutdown of retinal input to the cortex, e.g. by suppression of fixational eye movements [1], [9]. This is indicated by the residual sub-conscious processing of invisible stimuli, including the capacity to produce orientation-selective adaptation [10], negative afterimages [11], and Gestalt grouping [12]. Instead, MIB could be caused by a combination of low level mechanisms that trigger disappearance (such as adaptation [13], filling-in [14] and motion streak suppression [15]) with higher-level perceptual interpretation mechanisms that discard functionally inappropriate stimuli (such as depth ordering and surface completion [16]), or interpret transient activity changes as evidence for disappearance. Indirect physiological evidence was found for such a scheme [17] and for a related functional account of MIB [18].

In considering the processing underlying MIB it is useful to compare it with the simplest of the fading phenomena, Troxler fading [5]. In Troxler, a pattern of low contrast in the visual periphery disappears spontaneously with stable gaze, and its area filled-in by the surround. This filling-in property links Troxler fading to other types of filling-in phenomena [19], [20], although filling-in usually refers to a surrounding texture pattern, while Troxler fading refers to a uniform background. Troxler fading has been typically explained as the result of relative retinal stabilization leading to retinal and cortical adaptation [21], similar to that suggested for artificially stabilized images [22] and recently also for the monocular fading in binocular-rivalry (e.g. [23]).

There are notable phenomenological similarities between Troxler and MIB. Both do not occur at fixation, require steady gaze, increase with target eccentricity [20], [24], [25], decrease with target size [1], and tend to terminate with saccades or microsaccades [9], [26]. However, in all of the above dimensions, MIB is a stronger effect, showing disappearance even for high contrast, relatively large and salient patterns just off-fixation (1 deg) that do not disappear in Troxler (e.g. see Bonneh et al. 2001, results with static mask). These phenomenological similarities may lead to the idea that MIB is an enhanced form of the Troxler effect, possibly sharing common processes.

Gorea and Caetta [13] compared MIB and Troxler fading under the same conditions and proposed a low-level explanation and approach to the understanding of MIB. They suggested that MIB is (primarily) a combined effect of two well-known mechanisms: adaptation (of the local cortical response to the disappearing target) and prolonged inhibition of static by moving stimuli [27]. Adaptation was demonstrated by a reduction of perceived brightness of the target (which later disappears), and by increased suppression rates and durations in the first 10-15 sec of inspection. These were found to be similar in MIB and Troxler. Prolonged inhibition was indicated by threshold elevation in MIB relative to Troxler in a detection experiment (although this could also reflect a difficulty of detecting a temporal transient under the temporal clutter produced by the mask in their experiment). Based on these findings, these authors suggested that disappearance in both MIB and Troxler is determined by gain mechanisms that shift response relative to perceptual threshold, increasing or decreasing the probability of going below threshold. By a simple interpretation, this suggests that manipulations that shift this gain, such as changes in target contrast and mask speed should increase invisibility periods as well as decrease visibility periods or vice versa, with similar pattern of results for MIB and Troxler, despite the different amount of disappearance.

A very different pattern of results is predicted by the similarity of MIB to other types of perceptual bi-stability. In binocular rivalry and ambiguous motion for example, strengthening one stimulus, only shortens the dominance duration of the alternative percept, with a parallel increase of the alternation rate [28], [29]. This finding is well explained by a combined effect of local adaptation of stimulus-specific neuronal populations, competition between them, and noise [28], [30], [31]. The main difference between the two accounts is thus in the neural competition component.

A full and detailed comparison of the effect of stimulus strength on MIB and Troxler fading under the same conditions has not been reported till now, but there are some partial data, with somewhat contradicting results. In Troxler fading and other types of filling-in phenomena, an increased saliency of a target relative to its background is (usually) associated with increased time to fade ([24], [32], [33] (but see [20] for no such effect). In MIB on the other hand, higher proportion of disappearance was found with increased brightness of the targets in one study [1]. This effect was much smaller and variable and reversed at very low luminance in another study [20], possibly due to a difficulty in judging visibility of very dim targets. Interestingly, Hsu et al. [20] found a very similar pattern of results with a static mask (similar to Troxler, with additional random dot surround that may induce filling-in), with much lower disappearance values as expected and with large variability. Unfortunately, none of these studies reported the rate of disappearance events, which is a critical parameter in studies of bi-stability. Rate data were reported for MIB only [34] showing a slight increase in rate and decrease of percentage with target luminance contrast.

In the current study we conducted a straight forward comparison of the effect of stimulus strength on MIB and Troxler fading under the same conditions, to isolate common and different processes in these two phenomena. We found that both MIB and Troxler behave in a very lawful way as a function of target stimulus strength, but in a surprisingly opposite and different manner. We use this qualitative difference to interpret MIB as a combined effect of low-level adaptation mechanisms as in Troxler and cortical competition mechanisms analogous to the ones that are thought to underlie other bi-stable phenomena [28], [30], [31].

## Methods

### Subjects

Ten observers (5 females, ages 25–50) with normal or corrected-to-normal vision including two of the authors (YSB, THD) participated in the experiments. Most participants were experienced observers from previous MIB experiments.

The study and informed consent procedure were approved by the Weizmann Institute of Science Human Research Ethics Committee and a written informed consent was obtained from all participants.

### Apparatus

Stimuli were displayed on a 19” CRT monitor controlled by dedicated software developed by the first author running on a Windows PC. The video format was true color (RGB), 100-Hz refresh rate, 1280×1024 pixels resolution, and 25°×20° field of view. Luminance values were gamma-corrected to obtain linear luminance response with a range of 6.6–110 cd/m^2^. The viewing distance was 70 cm. All experiments were conducted in the dark.

### Stimuli

**Figure 1:**
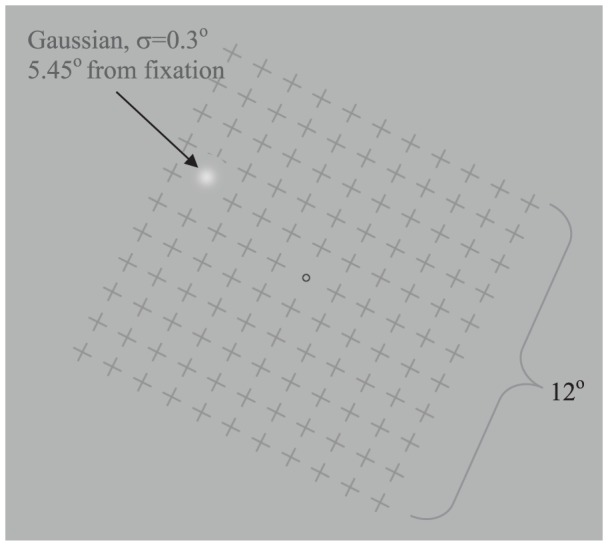
Example of stimuli used to measure MIB for different luminance contrast levels of a peripheral Gaussian target. Observers reported disappearance and reappearance of the Gaussian target by depressing buttons in 1 minute trials.

Stimuli consisted of a single Gaussian luminance patch (the target, σ = 0.3^o^) and a rotating mask (see example in Figure 1). The target was presented in the upper left quadrant (5.45^o^ of eccentricity) on a gray background (30 cd/m^2^) with one of seven different luminance contrasts, starting with 25% above background (peak luminance of 39.1 cd/m^2^) with increments of 0.15 log units. We use the term “contrast” to denote “luminance contrast” throughout the paper. The mask (when present) consisted of a square grid of 11×11 gray ‘+’ patterns (luminance of 13.3 cd/m^2^, width of 0.8^o^, spacing of 1.5 widths, total grid width of 12^o^). The mask rotated at one of 3 speeds: static (0), slow (0.023 cycle/sec equal to 43s cycles) and fast (0.37 cycle/sec equal to 2.7 s cycles). Both speeds were chosen with pilot experiments by the authors that verified high percentage of disappearance with the fast speed mask, similar to previous studies, and a clearly visible movement of the slow mask. There was a small black circle (0.27^o^ diameter) as a fixation point. Both the fixation point and the target were surrounded by a “protection zone” of background space (diameter:1.8^o^) to avoid local interaction with the mask.

### Procedure

All experiments consisted of several trials (duration: 1 min) involving continuous viewing of one of the above-mentioned stimulus configurations, which remained constant throughout the trial. Observers were instructed to fixate the central dot and depress and release a button to report disappearance and reappearance respectively of the target. Trials were initiated by the observer with a minimum of 5 sec period between trials. Stimuli varied between trials in target contrast and mask type (different speeds and no mask).

We performed two experiments. In experiment 1, five observers were tested only on the comparison between MIB (at fixed mask speed) and Troxler (no mask), with various different target contrasts. Each session (lasting for about half an hour) consisted of 2 repetitions of each of 28 trial types (7 contrast levels×2 stimulus conditions [MIB, Troxler]). The trial types were presented in random order. Each observer performed 2–4 sessions on different days, making each result the average of 4–8 samples per observer per trial type.

In experiment 2, the other five observers were tested in two further stimulus conditions in addition to MIB and Troxler. The four stimulus conditions were: fast mask (MIB), slow mask, static mask, and no-mask (Troxler), which were tested in separate blocks of trials in random order. Each block had the same 7 levels of contrast randomly presented in 1 minute trials, making 28 trials per session (about 30 minutes). Observers participated in 2–8 sessions (average of 5 per observer, only one observer with 2 sessions); to avoid sampling bias, results were first averaged across sessions individually for each observer, and then averaged across observers with equal weighting. The two sets of experiments differed in the order of presentation (mixed vs. blocked) and in the two additional stimulus conditions (static and slow mask).

### Data Analysis

Four parameters were computed for each trial: (1) percentage of total viewing time that the target was invisible (henceforth termed “percentage of time invisible”); (2) the average duration of target-invisible states (henceforth termed “mean invisible period”), (3) the average duration of target-visible states (henceforth termed “mean visible period”); (4) the number of disappearance events per minute (henceforth termed “transition rate”). These four parameters are inter-dependent, with two free parameters (i.e., two of the parameters are sufficient to derive the others, but one is insufficient). For example, the same percentage of time invisible can be produced by a few long invisible periods (at a low rate) or by many (high rate) short invisible periods.

Each of the four parameters was first averaged across trials, individually for each observer. A baseline value for each parameter and each observer was computed by averaging across contrast levels. The baseline values were subtracted from the values for each contrast to normalize the single-observer parameters. The normalized parameter values were then averaged across observers, and adjusted by adding the average (across observers) of the baseline values.

Statistical significance was verified by a two-way, repeated-measures within-observer analysis of variance (ANOVA). The two factors were contrast (7 levels) and mask condition (2 types in experiment 1, and 4 in experiment 2), both measured in the same observers.

## Results

In the two experiments, we manipulated the “strength” of the target (and the mask, experiment 2) and studied the effects on the dynamics of MIB and Troxler fading. There was no significant difference in the dependence on target contrast between experiments. Therefore, the data from both experiments (all 10 observers) were pooled for the common conditions of MIB and Troxler.

**Figure 2:**
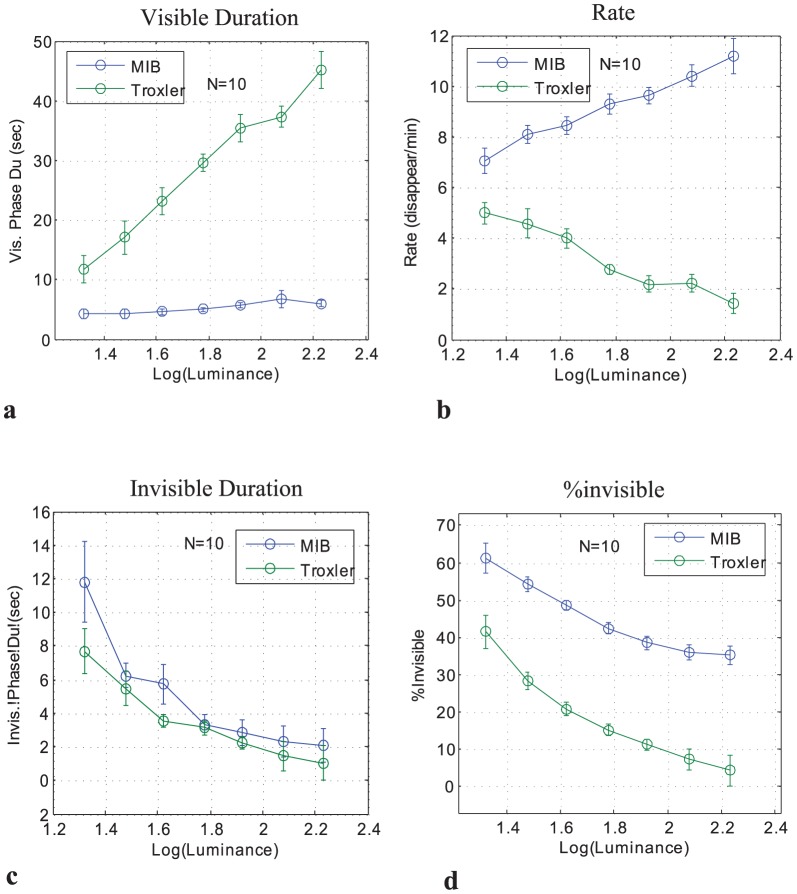
MIB and Troxler disappearance as a function of contrast (10 observers). (a) Mean visible periods. (**c**) Transition Rate (disappearance events per min). (**c**) Mean invisible periods. (**d**) Percentage of time invisible. Data were normalized individually for each observer before averaging across observers and adding the grand-average baseline (see Methods). Error bars denote SEM.

There was a strong dissociation between Troxler and MIB (Figure 2). The mean visible period was largely independent of contrast in MIB, but increased with contrast in Troxler (Figure 2a). The transition rate increased with contrast in MIB and decreased in Troxler (Figure 2b). The percentage of time invisible (Figure 2d) decreased with contrast for both MIB and Troxler, with higher values for MIB, and an insignificant difference between the slopes. Mean invisible period also decreased with contrast for both Troxler and MIB (Figure 2c), with no significant difference between them.

These findings were verified using a two-way (contrast, mask) repeated measures within-observer ANOVA, which showed (a) *mean visible periods*: a significant main effect of contrast, F(6,54) = 22.8, p = 0.0000, and mask (MIB vs Troxler), F(1,9) =  36.0, p = 0.0002, and a significant contrast×mask interaction, F(6,54) = 17.8, p = 0.0000; (b) *rate*: insignificant effect of contrast, F(6,54) = 0.63, n.s., a significant effect of mask, F(1,9) =  37.1, p = 0.0002, and a significant contrast×mask interaction, F(6,54) = 25.9, p = 0.0000; (c) *mean invisible period:* a significant main effect of contrast, F(6,54) = 8.0, p = 0.0000, a small effect of mask, F(1,9) =  7.4, p = 0.023, and insignificant contrast×mask interaction, F(6,54) = 1.71, n.s; (d) *percentage of time invisible:* a significant main effect of contrast, F(6,54) = 25.4, p = 0.0000, and mask, F(1,9) =  54.6, p = 0.0000, and insignificant contrast×mask interaction, F(6,54) = 1.06, n.s.

**Figure 3:**
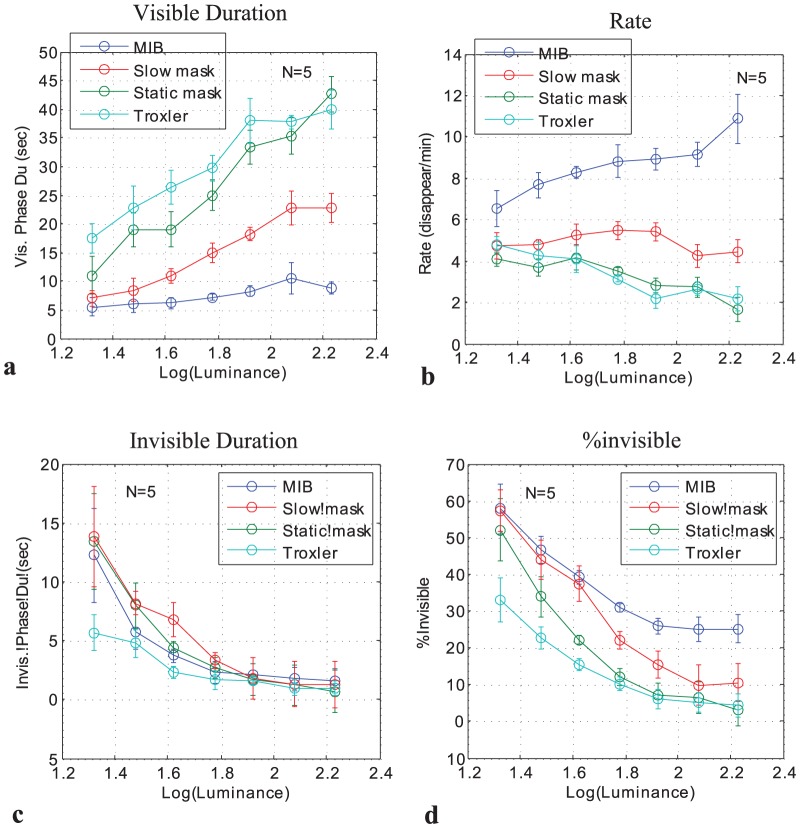
Disappearance as a function of contrast for Troxler (no mask), static mask, slow mask, and fast mask (5 observers). (a) Mean visible periods. (b) Transition Rate (disappearance events per min). (c) Mean invisible period. (d) Percentage of time invisible. Data were normalized individually for each observer before averaging across observers and adding the grand-average baseline (see Methods). Error bars denote SEM.

In experiment 2, we also manipulated the speed of the MIB mask (Figure 3). The results showed that the dependence on target contrast for the static mask was similar to Troxler (no mask), whereas slow mask yielded results in between no mask and fast mask. This was most notable for the transition rate, which increased with contrast for the fast mask, decreased with Troxler and static mask, and remained constant for the slow mask (Figure 3b). It was also clear for the visible period (Figure 3a), which increased with contrast, with a slope for the slow mask that was in between the slopes for Troxler (steep) and fast mask (approximately zero).

A two-way (contrast, mask) repeated measures within-observers ANOVA on the results of experiment 2 showed (a) mean visible periods: a significant main effect of contrast, F(6,24) = 21.5, p = 0.0000, and mask, F(3,12) =  8.62, p = 0.0025, and contrast x mask interaction, F(18,72) = 2.7, p = 0.0015; (b) rate: insignificant effect of contrast, F(6,24) = 0.69, n.s., a significant effect of mask, F(3,12) = 13.73, p = 0.0003, and contrast×mask interaction, F(18,72) = 3.73, p = 0.0000; (c) mean invisible period: a significant main effect of contrast, F(6,24) = 4.96, p = 0.002, insignificant effect of mask, F(3,12) =  1.91, n.s., and insignificant contrast×mask interaction, F(18,72) = 1.79, p = 0.06; (d) percentage of time invisible: a significant main effect of contrast, F(6,24) = 17.3, p = 0.0000, and mask, F(3,12) =  7.3, p = 0.0048, and insignificant contrast×mask interaction, F(18,72) = 1.72, p = 0.055.

**Figure 4:**
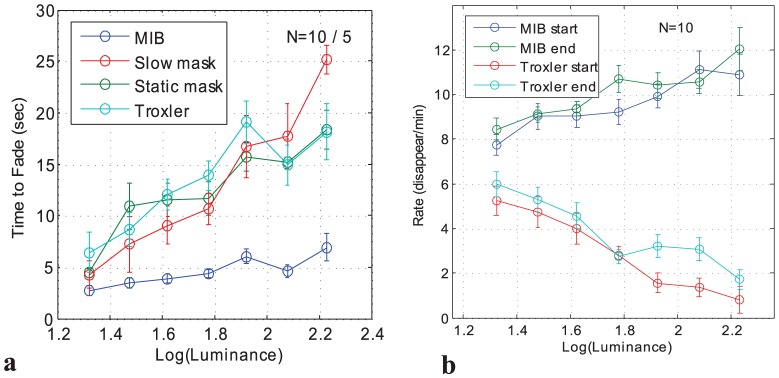
Two properties related to the effect of inspection time. **(a)** Time to fade (first disappearance) as a function of contrast for Troxler (no mask) and MIB (fast mask) (10 observers), as well as for static and slow mask, (5 observers). **(b)** Transition rate for the initial 15 sec part of the inspection time and the terminating 15 sec part. Data were normalized individually for each observer before averaging across observers and adding the grand-average baseline (see Methods). Error bars denote SEM.

We also computed two additional parameters related to the effect of inspection time (Figure 4). Figure 4a plots the time-to-fade (first disappearance in a trial). This measure is typically used to quantify the strength of the Troxler effect, although it does not take into account the dynamic bi-stable process. The time-to-fade showed a small effect of contrast in MIB, and a large effect (4–5 fold) in all other conditions (data for all 10 observers in MIB and Troxler, and for 5 observers for slow and static mask are superimposed). Figure 4b compares the transition rate during the first and last 15 sec of inspection. No significant difference was found, which implies that the transition rate does not change with inspection time. Similar results were found for the other parameters of visible and invisible periods and the percentage of time invisible (data not shown).

## Discussion

The current parametric manipulation of the “strength” (luminance contrast) of the disappearing target revealed qualitatively different effects on the dynamics of MIB and Troxler fading. The most striking difference (Figure 2a) was that the rate of Troxler fading events decreased with target contrast whereas the opposite was true for MIB (there were more MIB disappearance events with higher target contrasts). Another striking difference was that the mean visibility periods were largely independent of contrast for MIB, unlike Troxler (Figure 2b). Below we interpret these findings and discuss their implications for understanding the processes governing the dynamics of bi-stable perception.

### Invisible and visible periods vs. transition rate

The effects of contrast on mean invisible period (Figure 2c), mean visible period (Figure 2a), and transition rate (Figure 2b) are related as expected by their definition. For example, if the typical visible period does not change with contrast while the typical invisible period decreases, then the rate must increase.

The contrast dependence of the mean invisible period was similar for MIB and Troxler (Figure 2c), suggesting that the time it takes for a target to reappear is determined by processes not affected by the moving mask, thus common to MIB and Troxler. This can be (at least in part) a consequence of reappearances being triggered by small fixational eye movements (e.g., microsaccades), which do not depend on mask presence and speed [9], [26].

Mean visible periods were roughly constant with contrast for MIB (Figure 2a) but increased for Troxler, suggesting that MIB may involve an additional process that is largely independent of target contrast. The constant level of visible periods found in MIB could not reflect a “floor effect”, since measures much below this level were obtained in the same experiment for the invisible duration, and since the perceptual effect itself did not saturate as reflected in the changes of rate and invisible periods. The additional contrast-invariant component in MIB might be related to competition between the neuronal populations in visual cortex processing the target and the moving mask [35], [36].

### Relation to previous studies of MIB

In a previous study of MIB [1] the percentage of time invisible was found to increase with the luminance contrast of the target. Our current results (Figure 2d) show a different pattern, with a small decrease in the percentage of time invisible rather than increase. Among the main methodological differences we note the small eccentricity (1 deg) in the original study, which was not possible to repeat here to compare with a measurable Troxler effect (at 5.45^o^). Other differences include the presentation of 3 targets instead of one, and smaller target patterns with sharp edges rather than the Gaussian blobs used here. We suspect that the difference stems primarily from the difference in eccentricity which modulates the relative weight of the two suggested components involved in the process: contrast adaptation and neural competition. This results in a difference in the effect of contrast with eccentricity.

Another study which tested the effect of contrast on MIB [20], compared MIB with perceptual filling-in of the type first reported by Ramachandran and Gregory [4]. For MIB, the percentage of time invisible was similar in range to the current results (40–60%), for a similar range of target luminance contrast levels (10-fold and more). In comparison to the moderate decrease of the percentage of time invisible with contrast we observed (Figure 2d), they found a slight increase for brighter targets as well as for very dim targets (1 cd/m^2^ on a black background), possibly due to a difficulty in judging their visibility. In addition, they found the initial time-to-fade in MIB to be largely invariant to contrast (except from the very low luminance target) at about 3 sec, as compared to our moderate increase around 5 sec (Figure 4a). These moderate differences could be accounted for by our larger (∼3-fold) target size at roughly the same eccentricity, the different mask we used, as well as the larger variability in the Hsu et al data: standard errors 10-fold larger than our data, possibly due to a smaller sample (N = 5, half our sample size) and a larger inter-subject variability, which was minimized in our study by normalization (i.e., subtracting a baseline for each observer before averaging across observers), and by including observers experienced in MIB (see Methods, last paragraph).

More significant and striking is the discrepancy between the two studies in the static mask condition. While our static mask results were almost identical to the Troxler (no-mask) condition, showing decrease from 50% to almost zero invisibility with contrast, as well as increase from 5 sec to 20 sec in the initial time to fade, the Hsu et al study found around 30% invisibility and around 5 sec time-to-fade, largely independent of contrast. These differences are most likely due to the smaller target and the random dot mask in the Hsu et al study, which adds an additional component of perceptual filling-in. Hsu et al. [20] proposed that the non-intuitive contrast dependence (more MIB with higher contrast) is due to the effect of target-mask grouping: the more dissimilar the target is to the mask (e.g. differs in brightness), the less it groups with the mask, and the more it disappears. This explanation cannot account for the opposite pattern of results we obtained with a static mask and MIB (Figure 3b), and is inconsistent with the Bonneh et al. [1] results, where high luminance mask induced the strongest invisibility effect with high luminance targets. Given these discrepancies, the similarities between the effects of contrast in MIB and perceptual filling-in [20] cannot be used to make a strong claim about common mechanisms causing both effects. Instead, we propose that filling-in by a surrounding pattern (e.g. a proper static mask, but not Troxler) is an additional mechanism that affects disappearance. Taken together, the discrepancies between the current results and those from previous studies do not undermine our conclusions. The stimuli in the previous studies appear to have induced additional interactions beyond those processes revealed by our study.

The idea of two components involved in MIB was previously suggested by Gorea and Caetta [13] and is further supported by a detailed study of contrast detection and discrimination under invisible periods in MIB [37]. This study found both a sensitivity reduction indicative of inhibition and decisional criterion elevation indicative of an additional factor operating in MIB during invisibility [37]. In a following study, Gorea and Caetta [13] further specified these two components in terms of two gain modulation mechanisms: adaptation (of the neural population responding to the target), which causes a known response-gain change over time during continuous stimulation, and transient-to-sustained inhibition (of the response to the static target by the response to the moving mask), which causes a contrast-gain. These two components, together, can cause responses to the target to fall below perceptual threshold.

As discussed in the introduction, a simple implementation of this proposal implies a symmetric change of visible and invisible periods with changes in stimulus strength that alter these gains, for both MIB and Troxler fading. Our findings well match this pattern of results for Troxler but strongly deviate from it in MIB. This deviation is not related to adaptation over time, as we obtained a similar pattern of results when discarding the initial part of inspection (see Results), and thus should be explained by the dynamical properties of the proposed inhibition mechanism. In order to produce time-to-fade (or visibility period), which is (almost) invariant to contrast, one should assume inhibition which is proportional to contrast with a time course (to reach full inhibition effect) which is largely independent of contrast. Physiological evidence for contrast-dependent inhibition exists [38]. An alternative explanation, which is indicated by the similarity of the results to other types of bi-stable phenomena is related to competition and is discussed below.

### Relation between MIB and other bi-stable visual phenomena

Our findings link MIB (and Troxler fading) to two other bi-stable visual phenomena, for which parametric manipulations of the strength of the competing stimulus components produced analogous effects on the dynamics of perception: binocular rivalry (BR), [28] and a rotating structure-from-motion (SFM) sphere [29]. The perceptual dynamics in both phenomena are thought to emerge from the interplay between local adaptation of stimulus-specific neuronal populations, competition between them, and noise [28], [30], [31],with additional local spatial interactions in BR, not present in MIB due to the “protection zones” surrounding the target (see Methods). The analogy can be summarized in terms of Levelt's propositions for binocular rivalry (BR) and the deviations from these propositions at extreme values of stimulus strength [28], [29]. Unlike in BR, where stimulus dominance is quantified as visibility of one of the two stimuli, in MIB and Troxler we quantify the two (asymmetric) dominance states as target visibility and invisibility.

For MIB, the visible and invisible periods match Levelt's proposition 2: strengthening only one stimulus (target contrast) does not affect the duration of the corresponding percept (target visible period), but shortens the dominance duration of the alternative percept (target invisible period). By contrast, Troxler fading does not match that proposition. Exactly the same deviation as in Troxler also occurs in BR, when the contrast of the other (fixed-contrast) image is close to detection threshold [28]. Further, the results of our experiment 2 (manipulation of the “strength”, i.e., speed, of the mask) revealed that Levelt's proposition 2 also holds for the mask in MIB. Within the target contrast range (twofold increase), the mask speed does not affect the duration of the corresponding percept (invisible period), but shortens the duration of the alternative percept (visible period). Again, this symmetry of the effects of target and mask strength is analogous to BR [28] and SFM [29]. Finally, the effects of mask speed and target strength on the transition rate are analogous to SFM [28], [29]. This is true with respect to both matches and mismatches with Levelt's proposition 4: strengthening both stimuli will increase the alternation rate. In particular, when the strength of one of the two stimulus components is close to zero (i.e., static or absent mask: Troxler fading), the transition rate decreases with the strength of the other stimulus component (i.e., target contrast, Figure 2a, Troxler fading). The same is evident in SFM (Figure 5 of Klink et al., 2008).

Taken together, this comparison of results establishes common principles underlying MIB on the one hand and BR and SFM on the other hand. Such common principles are often suggested (e.g., [2], but direct evidence is scarce. Most authors have pointed to the similar (asymmetric) shapes of the distributions of the perceptual state durations in these phenomena (e.g., [2]. Measuring the perceptual dynamics under a full parametric manipulation of the relevant stimulus parameters, like the ones performed here, is more sensitive to establish such links, and our results are consistent with those for BR and SFM.

### Conclusion

In a detailed comparison of MIB and Troxler fading with respect to target contrast we found a lawful pattern of results with marked qualitative differences. Increasing target contrast increased (doubled) the rate of disappearance events in MIB, but shortened it to half in Troxler. On the other hand, MIB and Troxler did not differ in the mean invisibility periods which decreased with contrast. We interpret the results in terms of two processes involved in perceptual suppression: contrast adaptation, which is common to both MIB and Troxler, and neuronal competition, which is dominant in MIB, as well as in other bistable phenomena such as binocular rivalry.

## References

[1] Bonneh YS, Cooperman A, Sagi D (2001) Motion-induced blindness in normal observers. Nature 411: 798–801. 11459058 10.1038/35081073

[2] Blake R, Logothetis NK (2002) Visual competition. Nat Rev Neurosci 3: 13–21. 11823801 10.1038/nrn701

[3] Wilke M, Logothetis NK, Leopold DA (2003) Generalized flash suppression of salient visual targets. Neuron 39: 1043–1052. 12971902 10.1016/j.neuron.2003.08.003

[4] Ramachandran VS, Gregory RL (1991) Perceptual filling in of artificially induced scotomas in human vision. Nature 350: 699–702. 2023631 10.1038/350699a0

[5] Troxler D (1804) Uber das Verschwindern gegebener Gegenstande innerhalb unsers Gesichtskreises. In: Himly K, Schmidt JA, editors. Ophthalmologisches Bibliothek. Jena: Fromman. pp. 51–53.

[6] Simons D, Lleras A, Martinez-Conde S, Slichter D, Caddigan E, et al (2006) Induced visual fading of complex images. J Vis 6: 1093–1101. 17132081 10.1167/6.10.9

[7] Koch C (2007) The quest for consciousness: a neurobiological approach: Roberts & Company Publishers.

[8] Kim CY, Blake R (2005) Psychophysical magic: rendering the visible ‘invisible’. Trends Cogn Sci 9: 381–388. 16006172 10.1016/j.tics.2005.06.012

[9] Bonneh YS, Donner TH, Sagi D, Fried M, Cooperman A, et al (2010) Motion-induced blindness and microsaccades: cause and effect. J Vis 10: 22. 10.1167/10.14.22 PMC3075454 21172899

[10] Montaser-Kouhsari L, Moradi F, Zandvakili A, Esteky H (2004) Orientation-selective adaptation during motion-induced blindness. Perception 33: 249–254. 15109165 10.1068/p5174

[11] Hofstoetter C, Koch C, Kiper DC (2004) Motion-induced blindness does not affect the formation of negative afterimages. Conscious Cogn 13: 691–708. 15522627 10.1016/j.concog.2004.06.007

[12] Mitroff SR, Scholl BJ (2005) Forming and updating object representations without awareness: evidence from motion-induced blindness. Vision Res 45: 961–967. 15695181 10.1016/j.visres.2004.09.044

[13] Gorea A, Caetta F (2009) Adaptation and prolonged inhibition as a main cause of motion-induced blindness. J Vis 9 : 16 11–17. 10.1167/9.6.16 19761307

[14] Hsu LC, Yeh SL, Kramer P (2006) A common mechanism for perceptual filling-in and motion-induced blindness. Vision Res 46: 1973–1981. 16376963 10.1016/j.visres.2005.11.004

[15] Wallis TS, Arnold DH (2009) Motion-induced blindness and motion streak suppression. Curr Biol 19: 325–329. 19217295 10.1016/j.cub.2008.12.053

[16] Graf EW, Adams WJ, Lages M (2002) Modulating motion-induced blindness with depth ordering and surface completion. Vision Res 42: 2731–2735. 12450492 10.1016/s0042-6989(02)00390-5

[17] Libedinsky C, Savage T, Livingstone M (2009) Perceptual and physiological evidence for a role for early visual areas in motion-induced blindness. J Vis 9 : 14 11–10. 10.1167/9.1.14 PMC2654591 19271884

[18] New JJ, Scholl BJ (2008) “Perceptual scotomas”: a functional account of motion-induced blindness. Psychol Sci 19: 653–659. 18727780 10.1111/j.1467-9280.2008.02139.x

[19] Weil RS, Rees G (2011) A new taxonomy for perceptual filling-in. Brain Res Rev 67: 40–55. 21059374 10.1016/j.brainresrev.2010.10.004 PMC3119792

[20] Hsu LC, Yeh SL, Kramer P (2004) Linking motion-induced blindness to perceptual filling-in. Vision Res 44: 2857–2866. 15342229 10.1016/j.visres.2003.10.029

[21] Hunzelmann N, Spillmann L (1984) Movement adaptation in the peripheral retina. Vision Res 24: 1765–1769. 6533999 10.1016/0042-6989(84)90007-5

[22] Burbeck C, Kelly D (1984) Role of local adaptation in the fading of stabilized images. Journal of the optical society of America 1: 216–220. 6707778 10.1364/josaa.1.000216

[23] Kang MS, Lee SH, Kim J, Heeger D, Blake R (2010) Modulation of spatiotemporal dynamics of binocular rivalry by collinear facilitation and pattern-dependent adaptation. J Vis 10: 3. 10.1167/10.11.3 PMC2951267 20884498

[24] Livingstone MS, Hubel DH (1987) Psychophysical evidence for separate channels for the perception of form, color, movement, and depth. Journal of Neuroscience 7: 3416–3468. 3316524 10.1523/JNEUROSCI.07-11-03416.1987 PMC6569044

[25] Bonneh Y, Cooperman A (2003) Motion induced blindness is affected by head-centered and object-centered mechanisms. Journal of Vision 3: 221.

[26] Martinez-Conde S, Macknik SL, Troncoso XG, Dyar TA (2006) Microsaccades counteract visual fading during fixation. Neuron 49: 297–305. 16423702 10.1016/j.neuron.2005.11.033

[27] Breitmeyer B (1984) Visual masking: an integrative approach. New York: Oxford University Press.

[28] Brascamp JW, van Ee R, Noest AJ, Jacobs RH, van den Berg AV (2006) The time course of binocular rivalry reveals a fundamental role of noise. J Vis 6: 1244–1256. 17209732 10.1167/6.11.8

[29] Klink PC, van Ee R, van Wezel RJ (2008) General validity of Levelt's propositions reveals common computational mechanisms for visual rivalry. PLoS One 3: e3473. 18941522 10.1371/journal.pone.0003473 PMC2565840

[30] Noest AJ, van Ee R, Nijs MM, van Wezel RJ (2007) Percept-choice sequences driven by interrupted ambiguous stimuli: a low-level neural model. J Vis 7: 10. 10.1167/7.8.10 17685817

[31] Gigante G, Mattia M, Braun J, Del Giudice P (2009) Bistable perception modeled as competing stochastic integrations at two levels. PLoS Comput Biol 5: e1000430. 19593372 10.1371/journal.pcbi.1000430 PMC2700962

[32] Sturzel F, Spillmann L (2001) Texture fading correlates with stimulus salience. Vision Res 41: 2969–2977. 11704236 10.1016/s0042-6989(01)00172-9

[33] Welchman AE, Harris JM (2001) Filling-in the details on perceptual fading. Vision Res 41: 2107–2117. 11403794 10.1016/s0042-6989(01)00087-6

[34] Caetta F (2009) Interactions sensorielles et décisionnelles: une étude autour de la cécité induite par le mouvement: Université Paris Descartes, Paris, France.

[35] Donner TH, Sagi D, Bonneh YS, Heeger DJ (2008) Opposite neural signatures of motion-induced blindness in human dorsal and ventral visual cortex. J Neurosci 28: 10298–10310. 18842889 10.1523/JNEUROSCI.2371-08.2008 PMC2570589

[36] Donner TH, Sagi D, Bonneh YS, Heeger DJ (2013) Retinotopic Patterns of Correlated Fluctuations in Visual Cortex Reflect the Dynamics of Spontaneous Perceptual Suppression. J Neurosci 33: 2188–2198. 23365254 10.1523/JNEUROSCI.3388-12.2013 PMC3608931

[37] Caetta F, Gorea A, Bonneh Y (2007) Sensory and decisional factors in motion-induced blindness. J Vis 7: 4 1–12. 10.1167/7.7.4 17685800

[38] Polat U, Mizobe K, Pettet MW, Kasamatsu T, Norcia AM (1998) Collinear stimuli regulate visual responses depending on cell's contrast threshold. Nature 391: 580–584. 9468134 10.1038/35372

