# Correction: Motion-Induced Blindness and Troxler Fading: Common and Different Mechanisms

**DOI:** 10.1371/journal.pone.0101913

**Published:** 2014-06-27

**Authors:** The PLOS ONE Staff

There are several errors in Figures 2 and 3 and their respective figure legends. Please see the corrected figures and their legends below.

**Figure 2:**
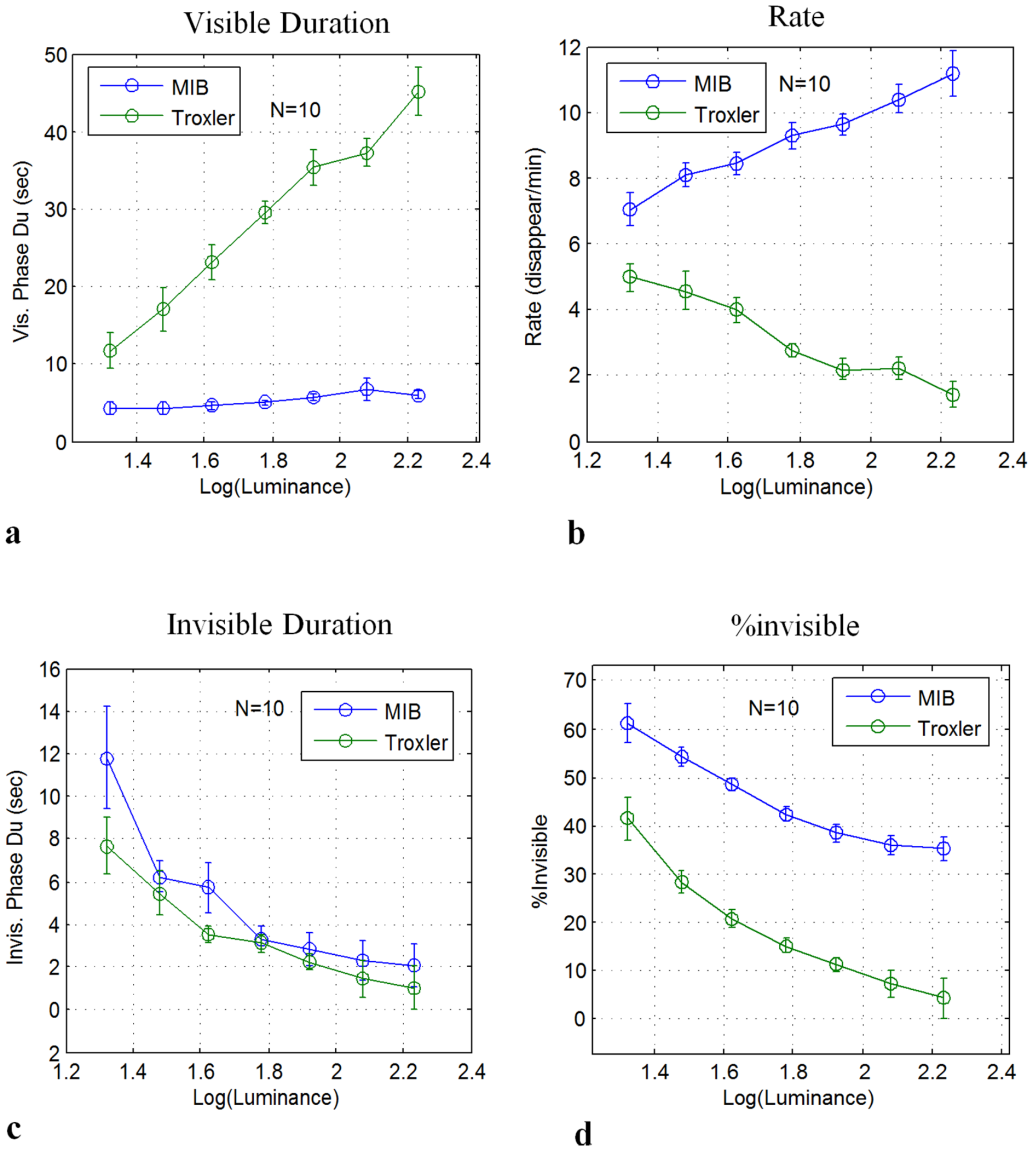
MIB and Troxler disappearance as a function of contrast (10 observers). **(a)** Mean visible periods. **(b)** Transition Rate (disappearance events per min). **(c)** Mean invisible periods. **(d)** Percentage of time invisible. Data were normalized individually for each observer before averaging across observers and adding the grand-average baseline (see Methods). Error bars denote SEM.

**Figure 3:**
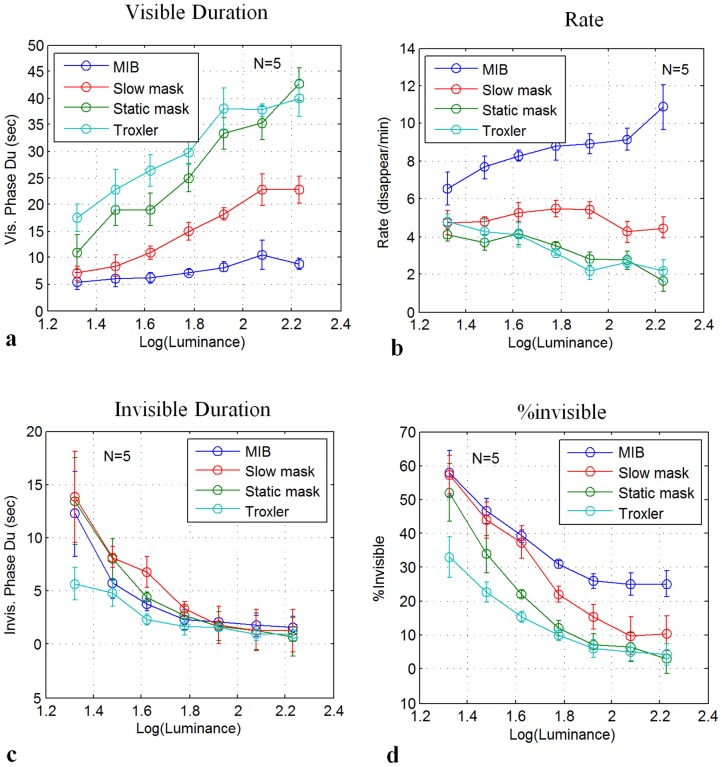
Disappearance as a function of contrast for Troxler (no mask), static mask, slow mask, and fast mask (5 observers). **(a)** Mean visible periods. **(b)** Transition Rate (disappearance events per min). **(c)** Mean invisible period. **(d)** Percentage of time invisible. Data were normalized individually for each observer before averaging across observers and adding the grand-average baseline (see Methods). Error bars denote SEM.
